# Virulence and Antifungal Susceptibility of *Microsporum canis* Strains from Animals and Humans

**DOI:** 10.3390/antibiotics10030296

**Published:** 2021-03-12

**Authors:** Chioma Inyang Aneke, Wafa Rhimi, Vit Hubka, Domenico Otranto, Claudia Cafarchia

**Affiliations:** 1 Dipartimento di Medicina Veterinaria, Università degli Studi “Aldo Moro”, 70010 Bari, Italy; chioma.aneke@unn.edu.ng (C.I.A.); wafa.rhimi@uniba.it (W.R.); domenico.otranto@uniba.it (D.O.); 2 Department of Veterinary Pathology and Microbiology, University of Nigeria, 410001 Nsukka, Nigeria; 3 Department of Botany, Faculty of Science, Charles University, 12801 Prague, Czech Republic; vit.hubka@gmail.com; 4 Laboratory of Fungal Genetics and Metabolism, Institute of Microbiology of the Academy of Sciences, 14220 Prague, Czech Republic; 5 Faculty of Veterinary Sciences, Bu-Ali Sina University, 6517658978 Hamedan, Iran

**Keywords:** dermatophytes, *Microsporum canis*, antifungal susceptibility testing, virulence enzymes, phospholipase, catalase, thermotolerance

## Abstract

The enzymatic and antifungal profiles of dermatophytes play an important role in causing infections in humans and animals. This study aimed to assess the virulence factors produced by *Microsporum canis* strains, in vitro antifungal profile and the relationship between virulence, antifungal profile and occurrence of lesions in animals and humans. A total of 100 *M. canis* strains from humans with *tinea corporis* (*n* = 10) and from animals presenting (*n* = 64) or not (*n* = 26) skin lesions was employed to evaluate phospholipase (Pz), hemolytic (Hz), lipase (Lz), catalase (Ca), and thermotolerance (GI) activities. In addition, in vitro antifungal profile was conducted using the CLSI broth microdilution method. A statistically significant difference (*p* < 0.05) in Lz and Ca values was revealed among strains from hosts with and without lesions. Voriconazole, terbinafine, and posaconazole were the most active drugs followed by ketoconazole, griseofulvin, itraconazole, and fluconazole in decreasing activity order. The significant positive correlation between azole susceptibility profile of *M. canis* and virulence factors (i.e., hemolysin and catalase) suggest that both enzyme patterns and antifungal susceptibility play a role in the appearance of skin lesions in animals and humans.

## 1. Introduction

*Microsporum canis* is one of the major pathogenic and most prevalent dermatophytes of domestic animals with a zoonotic potential that may cause *tinea capitis* and *tinea corporis* in humans [1,2,3,4,5,6]. Clinical presentation of this infection is characterized by multifocal alopecia and scaly, circular lesions (usually referred to as “ringworm”), similar to that caused by other dermatophytes or other skin diseases [7]. The pathogenesis of this infection is usually associated with the secretion of virulence factors like keratinase or activation of genes involved in keratinolytic activity (e.g., of the sulfite efflux pump, cysteine dioxygenase, endoprotease, and exoproteases) [8,9]. However, enzymes secreted by dermatophytes during infections differ according to the type of lesions and dermatophyte species [9,10,11]. Interestingly, keratinolytic activity seems to be correlated with the appearance of skin lesion, irrespective of the species of dermatophyte. 

Other enzymes may occur but varies according to the species and hosts and pathologies [9]. For example, protease production may be associated with human infections caused by *Trichophyton mentagrophytes*, DNase by *T. verrucosum* and hemolytic activity by all dermatophytes isolated from human infections [9]. Phospholipase and lipase are involved in degrading the infected tissue components, irrespective of the dermatophyte’s species [9,12,13]. Phospholipase secreted (Pz) might cause tissue damage through the hydrolysis of phospholipids and degradation of cell membranes [14], while lipase (Lz) is essential in the initial stages of dermatophyte infection through invasion of the stratum corneum in animals [13].

Other virulence factors such as catalase (Ca), hemolytic factors (Hz) and thermotolerance (GI) have been investigated in yeasts and molds causing human and animal skin infections [15], but not in *M. canis.*

Current treatment against dermatophytoses in pet animals includes the use of oral antifungals such as itraconazole (ITZ), ketoconazole (KTZ), terbinafine (TER), topical antifungal therapy, routine cleaning of the premises, limited confinement and treatment monitoring [7,16], whereas for humans, fluconazole (FLZ) represents the first line therapy in many European countries [17]. Azole resistance phenomena in *M. canis* is rarely reported, [18] although clinical evidence suggest that some of the infections are difficult to treat with azoles or TER [18,19]. Although a standardized antifungal susceptibility testing protocol for *M. canis* has not been developed [20], many studies on the antifungal therapy suggested that *M. canis* have low susceptibility to ITZ or FLZ [18,21,22]. Interestingly, the above findings were observed in *M. canis* isolates from humans and animals with skin lesions [18,21,22], and most likely, might be due to an acquired azole resistance phenomenon which resulted from the recurrent use of these drugs for the treatment of infections [7,23]. Nonetheless, the low antifungal susceptibility associated with the low expression of virulence factors in yeasts might explain the positive outcome of lesions on infected animals [15]. Therefore, since no studies have been conducted on the relationship between virulence factors and antifungal resistance phenomena in *M. canis* strains, the aims of the study were: (i) to assess the capability of *M. canis* strains isolated from animals with and without skin lesions and humans with *tinea corporis* in producing virulence factors; (ii) to evaluate their in vitro antifungal susceptibility to ITZ, KTZ, voriconazole (VOR), posaconazole (PSZ), TER, FLZ and griseofulvin (GRI) using a modified CLSI broth microdilution method; and (iii) to assess the relationship between the virulence factors and the antifungal susceptibility of *M. canis* strains from different hosts and pathology.

## 2. Results

### 2.1. Strain Identification

A total of 100 *M. canis* strains from humans with *tinea corporis* (*n* = 10), animals with (*n* = 64) and without (*n* = 26) skin lesions showed phenotypic features typical for *M. canis* (i.e., rapid growth, colony reverse in shades of yellow or orange, thick-walled spindle-shaped macroconidia in sporulating strains) and were also identified by improved molecular diagnostic assay, as previously reported [24]. In addition, amplification of the internal transcribed spacer of nuclear ribosomal DNA (ITS rDNA) region, a gold standard for dermatophyte identification, confirmed identification in 62 selected strains. There were only two ITS genotypes among the examined strains. The genotypes were deposited into the European Nucleotide Archive (ENA) database under the accession numbers LR989561 and LR989562. The first genotype (G1, GenBank accession: LR989561) was present in 59 strains and the second genotype (G2, GenBank accession: LR989562) only in 3 strains. Genotype G1 was identical to the M. canis ex-type strain CBS 496.86 (MH861991) and the genotype G2 had one substitution (*n* = 388) in the 5.8S region compared to G1 (Supplementary Table S1). 

### 2.2. Virulence Attributes

The presence of virulence factors of *M. canis* strains from animals with and without lesions and humans are reported in Table 1 and Supplementary Figure S1. 

A total of 94 (94%) and 92 (92%) *M. canis* strains showed Pz and Hz, respectively. All *M. canis* (100%) showed Lz, Ca, and GI. No statistically significant difference was found between Pz and Hz values in *M. canis* strains of different origin, (i.e., animals with, without lesions and humans). 

Statistically significant difference in the Lz values were recorded among *M. canis* strains from animals with or without lesions and humans, being lower (i.e., high activity) in strains from animals without lesions (Lz = 0.6) than those from animals with lesions (Lz = 0.8) and from humans (Lz = 0.7) (Table 1). 

All *M. canis* strains exhibited Ca with a statistically higher number of strains producing high Ca values in animals with lesions and humans with *tinea corporis* than that observed in animals without lesions (Table 1). The growth of *M. canis* colonies at 28 °C was better than at 35 °C (*p* < 0.05). Thermotolerance of *M. canis* strains varied according to the *M. canis* origin. A higher number of *M. canis* strains presenting low thermotolerance was observed in animals with lesions and humans with *tinea corporis.*

### 2.3. In Vitro Susceptibility Testing

VOR, TER, and PSZ were the most active drugs against *M. canis* strains, followed by KTZ GRI, ITZ, and FLZ in decreasing activity order (Table 2).

The minimum inhibitory concentrations (MIC) data varied according to the origin of *M. canis* strains for all the drugs tested (Table 2). In particular, the ITZ mean MIC values of *M. canis* from animals without skin lesions were statistically lower than those observed in strains from animals with lesions. Furthermore, ITZ, FLZ, and KTZ MIC ranges of *M. canis* from animals without skin lesions were lower than those observed in strains from animals with lesions and humans with *tinea corporis.* MIC_90_ values for ITZ, POS, TER, and FLZ were higher in strains from animal with lesions than those observed in animals without lesions (Table 2).

The VOR, PSZ, and GRI MIC ranges of *M. canis* from humans (0.008–0.06, 0.008–0.125, 0.125–0.5 μg/mL, respectively) were lower than those in animals with (0.008–0.5, 0.008–2, 0.06–2 μg/mL, respectively) and without skin lesions (0.008–0.5, 0.008–2, 0.125–2 μg/mL, respectively).

### 2.4. Association between Virulence Attributes and MIC Values in M. canis Strains from Different Origin

Correlation data between virulence factors and MIC data of seven antifungals in *M. canis* isolated from animals and humans with and without skin lesions are shown in Table 3.

No statistical differences in the production of virulence factors were observed between strains with low (MIC > MIC_90_) and high (MIC ≤ MIC_90_) KTZ, POS, and TER susceptibilities, regardless of the occurrence of lesions. In strains from animals with skin lesions, the Hz and the Ca activities varied according to the azole, GRI and TER susceptibilities. Particularly, a statistically lower Hz (higher activity, *p* < 0.05) was observed in low VOR and FLZ susceptible strains than those in high susceptible ones. A statistically lower Ca (*p* < 0.05) was registered in low KTZ, VOR, PSZ, and TER susceptible strains than in high susceptible ones. In animals without skin lesions, a statistically lower (*p* < 0.05) Lz (higher activity) was registered in *M. canis* strains with low susceptibility to ITZ and a statistically lower Ca in those with low susceptibility to GRI. No statistically significant differences were observed for the correlation between thermotolerance and drug susceptibilities of *M. canis* strains.

## 3. Discussion

Data presented in this study show that *M. canis* strains isolated from humans and animals with and without skin lesions produce different virulence factors with a variable antifungal profile which might be linked to occurrence of skin lesions. The pathogenesis of *M. canis* infections is due to its virulence factors and host receptivity. Particularly, it is well known that keratinase or gene expression associated with keratinolytic activities is one of the most important virulence factor produced by some of the dermatophytes [8], and this might explain their pathophysiological mechanisms which is mainly linked to their survival on host tissues and the appearance of skin lesions [11,25,26].

While the dermatophyte’s keratinolytic activities are well investigated in different studies [11,24], it is still not known whether there is a correlation between other enzyme profiles and the clinical manifestation of the dermatophyte infections. In this study, at least 92% of *M. canis* strains were able to produce phospholipase, hemolysin, catalase, and lipase regardless of their origin and clinical manifestation (with or without lesion) suggesting that these enzymes are integral part of the enzymatic machinery of *M. canis* and might favor its survival on host’s tissues. Particularly, the finding that the production of phospholipase did not vary according to the origin of *M. canis* suggests that phospholipases might not be linked to the occurrence of skin lesions and might be responsible for the colonization of host cells. These results are in agreement with those previously reported [9,11] thus, confirming the importance of these enzymes in the first phase of *M. canis* infection (i.e., colonization). 

In addition, the findings that all *M. canis* strains are catalase producers suggests that *M. canis* is able to destroy H_2_O_2_ radicals, by reducing the probability of converting the peroxide produced by phagocytic cells into more potent and microbicidal reactive oxygen intermediates [27]. Interestingly, the high value of catalase activities observed in strains from animals and humans with skin lesions might suggest that this enzyme might have a role in the occurrence of skin lesions. 

Furthermore, the presence of high catalase producing *M. canis* strains in animals without lesions might suggest that the infection was in its early stage and the lesions had not yet appeared as at the time of sampling. Since studies on the ability of *M. canis* strains to produce this enzyme are very limited, future studies will be useful to ascertain whether catalase could be a target enzyme necessary to understand the evolution of the disease caused by *M. canis*. As for lipase activity, the finding of this enzyme in all *M. canis* strains regardless of their origin might suggest that it is correlated with *M. canis* colonization by breaking the lipid surface layer during the initial phase of infection, as previously observed [28]. These results are in agreement with other studies in which all *M. canis* strains from both healthy and symptomatic animals showed lipase activities [10,29] but differ from a study of *M. canis* from humans [30], thus suggesting that the host species receptivity might affect the pathogenesis of *M. canis* infection. Interestingly, the finding that the reduction in the lipase activity affects the appearance of skin lesions both in humans and animals might suggest that the lipase activity of *M. canis* has a protecting role on hosts tissue. 

Few studies on lipase production by dermatophyte have been published and the conclusions are contradictory [10,11,29,31,32]. In particular, Lopez-Martinez et al. [29] observed that all dermatophytes, including *M. canis*, displaying lipase activity were associated with chronic dermatophytosis. Oppositely, Viani et al. [32] showed no significant difference in lipase activity between *M. canis* strains isolated from acute and chronic infections. Additionally, a higher level of lipase activity in *M. canis* than in *T. mentagrophytes* isolates was observed previously, showing a species-specific enzyme profile associated to the occurrence of lesions on animals’ skin [10,30]. The above results and that obtained in this study suggest the need of future studies on the specific pathway for this enzyme for *M. canis* infection mainly using a more accurate method for lipase quantitative evaluation (i.e., volumetry, spectrometry, radioactive assay, immunoassay, conductimetry, chromatography, and biosensors) [33].

It is noteworthy that 92% of *M. canis* strains from hosts with or without skin lesions showed hemolytic activity suggesting that *M. canis* produces these enzymes for a survival strategy during infection. Hemolysins produced by dermatophytes may play an important role in the balance between the host’s cellular immunity and the ability of the fungus to diminish the immune response [34]. The result is in agreement with other studies that also found 100% hemolytic activity in *T. mentagrophytes* and *M. canis* strains from humans with and without skin lesions [30]. However, contrasting results have been reported and most likely, might be due to the methods used for the evaluation of hemolytic activities [11,34].

The finding that low thermotolerance of *M. canis* was mainly observed in strains from animal with lesions and humans with *tinea corporis* might suggest that this activity is linked to the occurrence of skin lesions, as previously reported [35]. However, since low thermotolerance were observed also in animals without lesions, the role of thermotolerance in causing skin lesions should be further investigated. As regards the in vitro susceptibility testing, in this study, VOR, TER, PSZ had the highest antifungal activity against *M. canis* strains from all groups, suggesting that they might be drugs for an effective treatment of dermatophytosis in both humans and animals [18]. In particular, it has been shown that VOR has an effective in vitro and in vivo activity against *M. canis* specifically in the range of 0.01 to 0.5 μg/mL [36,37,38] and the PSZ has been demonstrated to show high fungicidal activity against all *Microsporum* spp. [39].

Finally, the finding of low activities of ITZ and FLZ regardless of the origin of strains is most likely due to the common employment of these drugs to treat or prevent animal and human dermatophyte infections and might suggest that they are not an optimal choice for long term therapy [18,40]. Since the MIC data varied among *M. canis* strains from hosts with and without skin lesions, their association with virulence factors may be linked to the appearance of visible symptoms in *M. canis* infections in animals and humans. In this study, the significant positive correlation of VOR, FLZ, KTZ and VOR, PSZ, TER, FLZ high MIC values with Hz and Ca, respectively, in *M. canis* strains from hosts with skin lesions suggests that both the enzyme patterns and antifungal susceptibility might play a pivotal role in the appearance of skin lesions. Furthermore, the high ITZ and GRI susceptibility and the Lz and Ca activities, respectively, in animals without skin lesions might be the cause of absence of lesions both in animals and humans. In conclusion, the results of the present study indicate that clinical isolates of *M. canis* from different human and animal origins produce enzymes with different levels of activities. The profile of enzymes might be dependent upon factors related to the host. The relationship between each enzyme and the occurrence of skin lesions in animals and humans or asymptomatic animal varies. Furthermore, only catalase and lipase activities seem to be well correlated with the appearance of skin lesions. VOR, TER, PSZ are the most active drugs for the treatment of both humans and animals dermatophytosis. Additionally, the antifungal profile associated with enzyme pattern are important in explaining the evolution of pathology caused by *M. canis*.

Future studies aimed to understand better the association between virulence factors and antifungal resistance at genetic and molecular levels will assist in developing new therapeutic strategies for the effective treatment of *M. canis* infections.

## 4. Materials and Methods

### 4.1. Source of Strains and Their Identification

A total of 100 *M. canis* strains from humans with *tinea corporis* (*n* = 10), animals with (*n* = 64) and without skin lesions (*n* = 26) were employed in this study and grouped on the basis of their origin and presence of lesions.

The strains were identified phenotypically based on colonial morphology, microscopic features showing the hyphae, macroconidia, microconidia [41] and by an improved molecular diagnostic assay, as previously reported [24]. Isolates were deposited in the fungal collection of the Department of Veterinary Medicine at the University of Bari (Italy). Prior to testing, each strain was subcultured twice onto Potato Dextrose agar (PDA, Liofilchem, Roseto degli Abruzzi, Italy) plates at 28 °C for 14 days to ensure strain purity and viability.

Correct identification was confirmed on 62 strains which were selected based on their virulence profile and morphology. ArchivePure DNA yeast and Gram2 + kit (5PRIME) was used to isolate genomic DNA from 7 days old colonies grown on malt extract agar (MEA: HiMedia, Mumbai, India) as described by Hubka et al., [42]. Forward primers ITS1F (5′-CTTGGTCATTTAGAGGAAGTAA), ITS1 (5′-TCCGTAGGTGAACCTGCGG) or ITS5 (5′-GGAAGTAAAAGTCGTAACAAGG) in combination with reverse primers ITS4S (5′-CCTCCGCTTATTGATATGCTTAAG) or NL4 (5′-GGTCCGTGTTTCAAGACGG) were used for amplification of the ITS rDNA region (ITS1-5.8S-ITS2 cluster). PCR conditions followed a protocol previously described [43]. The obtained DNA sequences were compared with those derived from the ex-type or reference strains and unique genotypes were deposited into the European Nucleotide Archive (ENA) database under the accession numbers LR989561 and LR989562.

#### Enzymatic Activity

Colonies inoculated onto PDA, incubated at 28 °C for 14 days were harvested and transferred into sterile saline solution and adjusted to an optical density of 2.4 McFarland which is equivalent to 1–5 × 10^6^ colony forming units (CFU)/mL as validated by quantitative plate count of CFU on PDA. Then, 50 μL of each strain suspension was cultured on special media for testing the enzymatic activities. The culture in PDA was used as a negative control. Each test was performed in duplicate. The results were expressed as the average of the two obtained values. The student *t*-test was used to evaluate the differences among enzymatic activities mean values within different groups and Chi-square test was used to evaluate the differences among the number of isolates presenting enzymatic activities within different groups. A value of *p* < 0.05 was considered statistically significant. 

### 4.2. Phospholipase Activity 

The phospholipase activity (Pz) was assessed using the egg-yolk plate method, as previously reported [44]. Briefly, a total of 50 µl of *M. canis* suspension was transferred to egg-yolk plates (peptone 1%, dextrose 2%, calcium chloride 0.05%, sodium chloride 5.73%, agar 2%, and egg yolk 5%) and incubated at 28 °C for 14 days. The formation of a clear halo zone surrounding the colonies indicated phospholipase production. The production of phospholipase (Pz) was expressed as a ratio of diameter of a colony to total diameter plus zone of precipitation [44].

### 4.3. Detection of Hemolytic Activity 

Columbia blood agar base supplemented with sheep blood was utilized for the detection of hemolytic activity (Hz) [34]. A total of 50 μL of each strain suspension was cultured in the above medium and incubated at 28 °C for 14 days. A transparent zone of clearance around the colony indicated complete hemolysis. The Hz was expressed as a ratio of diameter of a colony to total diameter plus zone of precipitation [44].

### 4.4. Lipase Activity 

The lipase activity (Lz) was assessed, as previously reported [31]. Briefly, 50 μL of each strain suspension was cultured in a sterile petri disk containing a lipid medium (i.e., peptone 1%, sodium chloride 5%, calcium chloride 0.01%, and agar 2%, plus 1% tween 80) and incubated at 28 °C for 14 days. A clear halo zone of precipitation around the colony indicated lipase production [31]. The production of lipase (Lz) expressed as a ratio of diameter of a colony to total diameter plus zone of precipitation [44].

### 4.5. Catalase Activity 

Determination of catalase activity (Ca) was performed using a semiquantitative assay. In brief, 50 μL of each strain suspension was cultured in a screw-cap tube containing PDA medium and incubated at 28 °C for 14 days. Then, 1 mL of a freshly prepared mixture of 10% tween 80 (Sigma–Aldrich, St Louis, MO, USA) and 30% hydrogen peroxide (Proquimios, Rio de Janeiro, Brazil) were added and the column of bubbles was measured using a millimetric ruler after five minutes at room temperature. Uninoculated medium was used as a negative control. Strains were classified as low or high catalase producers if the size of the column of bubbles was less or higher than 45 mm, respectively.

### 4.6. Thermotolerance Determination 

The thermotolerance (GI) of *M. canis* was assessed as previously reported for *Sporothrix* spp. with few modifications [35]. Briefly, *M. canis* strains was cultured on PDA agar and incubated at 35 °C and 28 °C for 14 days. The diameter of the strains were measured with a millimeter ruler and the percentage inhibition of growth (GI%) was calculated using the formula [1−(D35/D28)] × 100, where D28 and D35 are the colony diameters at 28 °C and 35 °C, respectively [45]. The strains with GI% ≥ 50% were. classified as having low thermotolerance and the strains with GI% < 50% were classified as having high thermotolerance.

### 4.7. The Antifungal Susceptibility Testing

The antifungal susceptibility of the *M. canis* strains was tested using the reference CLSI BMD M38-A2 assay with some modifications [46]. A broth microdilution assay was performed in RPMI 1640 medium (Sigma–Aldrich, St Louis, MO, USA) with L-glutamine but without sodium bicarbonate and buffered with 0.165M morpholine propane sulfonic acid (MOPS) (Sigma–Aldrich, St Louis, MO, USA) at pH 7.0. Antifungal drug stocks and the inoculum suspensions were prepared, as described below.

### 4.8. Antifungal Agents

The following drugs were obtained in their standard powder state: TER and GRI (Sigma–Aldrich, Milan, Italy), KTZ and VOR (Novartis, Basel, Switzerland), FLZ (Pfizer, Kent, UK), ITZ (Janssen Research Foundation, Beerse, Belgium), and PSZ (Schering Plough Research, Kenilworth, NJ, USA). Stock solutions of FLZ (10 mg/mL), KTZ (10 mg/mL), ITZ (10 mg/mL), VOR (10 mg/mL), PSZ (10 mg/mL), TER (10 mg/mL), and GRI (50 mg/mL) were prepared by dissolving the powders in their respective solvents. FLZ was dissolved in distilled water while the other compounds were dissolved in 100% dimethyl sulfoxide (DMSO, Sigma–Aldrich, Milano Italy). A solution of 100% DMSO (128 µg/mL) was used as control in order to evaluate any potential effect of DMSO. The stock solutions were stored at −20 °C until use.

### 4.9. Inoculum Preparation

Inocula were obtained from 14-day-old *M. canis* cultures growing on Potato Dextrose Agar (PDA, Liofilchem, Roseto degli Abruzzi, Italy) and incubated at 28 °C, as previously reported [46]. Briefly, mature colonies were submerged with approximately 5 mL of sterile saline solution (0.85% *w*/*v*) and the surface was scraped with the tip of a Pasteur pipette. The resulting mixture was transferred into a 10 mL sterile tube. The mixture of conidia and hyphal fragments were allowed to sediment for 15 min at room temperature and the supernatant was collected and filtered using sterile filter paper (Whatman filter, model Grade 40, pore size 8 µm), which retains hyphal fragments. The density of the filtered suspension was adjusted to an optical density of 2.4 McFarland, as previously reported [46].

### 4.10. In Vitro Susceptibility Testing

The concentration of each antifungal drug ranged from 0.008 to 16 µg/mL with the exception of FLZ and GRI, whose concentration ranged from 0.06 to 128 µg/mL. A total of 100 µL of each antifungal drug concentration was transferred into a 96-well microtiter plate and 100 µL of inoculum solution was added. Visual reading of plates was performed after 3 days of incubation at 30 ± 2 °C. The MIC of each strain was defined as the lowest concentration of the antifungal producing a predominant decrease in turbidity (i.e., 100% of inhibition) when compared to the control growth, as previously described [46,47,48]. Each plate was duplicated, and each drug dilution tested in duplicates in each plate. Quality control strains (*Candida parapsilosis* ATCC 22019 and *Candida krusei* ATCC 6258; American Type Culture Collection, Manassas, VA, USA) were included to ensure accuracy of the drug dilutions and reproducibility of the results. Data obtained were reported as MIC ranges, MIC mean value (MICm), and MIC at which 50% (MIC_50_) and 90% (MIC_90_) of the strains were inhibited.

Both on-scale and off-scale results were included in the analysis. The low and high off-scale MICs were converted as the lowest MIC or the highest MIC, respectively. In the absence of clinically validated MIC breakpoints for antifungal susceptibility testing of *M. canis*, the strains were divided as low or highly susceptible when their MIC values were >MIC_90_ or ≤MIC_90_, respectively. The student *t*-test was used to evaluate the differences among MIC mean values of different antifungal agents within different groups. A value of *p* < 0.05 was considered statistically significant.

## Figures and Tables

**Table 1:** Number (Positive/Total) and percentage (%) of *Microsporum canis* strains presenting phospholipase (Pz), catalase (Ca), lipase (Lz), hemolytic (Hz) activities, and thermotolerance (GI) according to their origin (animals with and without lesions and humans). The Pz, Lz, and Hz activities were expressed as mean values with standard deviation (sd) in bracket. Ca and GI were reported as the number and percentage (%) of strains producing high values.

*M. canis* Source	Phospholipase		Catalase		Lipase		Hemolysis		Thermotolerance		
	Pos/Tot (%)	Mean Pz (sd)	Pos/Tot (%)	Number of Strains (%) with High CA Value (>45 mm)	Pos/Tot (%)	Mean Lz (sd)	Pos/Tot (%)	Mean Hz (sd)	Diameter (mm) Mean at 28 °C (sd)	Diameter (mm) Mean at 35 °C (sd)	Number of Strains (%) with High GI% Value (>50)
Animals without lesions	24/26 (92.3)	0.4 (0.2)	26/26 (100)	2/26 (7.7) ^a b^	26/26 (100)	0.6 (0.04) ^c d^	20/26 (77)	0.6 (0.3)	73.7 (8.9)	45 (5.7)	6/26 (23) ^e^
Animals with lesions	60/64 (94)	0.5 (0.2)	64/64 (100)	40/64 (62.5) ^a^	64/64 (100)	0.8 (0.4) ^c^	62/64 (97)	0.7 (0.1)	79.2 (4.4)	36.1 (6.2)	48/64 (75) ^e^
Humans	10/10 (100)	0.5 (0.1)	10/10 (100)	10/10 (100) ^b^	10/10 (100)	0.7 (0.03) ^d^	10/10 (100)	0.7 (0.1)	79 (4.2)	35.4 (7.0)	8/10 (80)
Total	94/100 (94)	0.5 (0.2)	100/100 (100)	52 (52)	100/100 (100)	0.8 (0.4)	92 (92)	0.6 (0.2)	77.7 (6.2)	38.3 (7.2)	64/100 (64)

^a,b,e^ Chi-square test—statistically significant differences (*p* < 0.05) were marked with the same letters. ^c,d^ Student’s *t*-test—statistically significant differences (*p* < 0.05) were marked with the same letters. For Pz, Lz and Hz, lower values represent higher activity. GI% > 50 represents low thermotolerance.

**Table 2:** Itraconazole (ITZ), ketoconazole (KTZ), voriconazole (VOR), posaconazole (PSZ), terbinafine (TER), fluconazole (FLZ) and griseofulvin (GRI) MIC data (μg/mL) of *Microsporum canis* strains from humans with *tinea capitis* and animals with and without skin lesions.

Drugs	Animals without Skin Lesions (*n* = 26)				Animals with Skin Lesions (*n* = 64)				Humans (*n* = 10)				Total (*n* = 100)			
	MIC Range	MIC_50_	MIC_90_	MIC Mean (sd)	MIC Range	MIC_50_	MIC_90_	MIC Mean (sd)	MIC Range	MIC_50_	MIC_90_	MIC Mean (sd)	MIC Range	MIC_50_	MIC_90_	MIC Mean (sd)
ITZ	0.03–2	1	1	0.9 (0.6) ^a^	0.125–8	2	4	2.4 (2.3) ^a^	0.25–4	1	4	1.5 (1.5)	0.03–8	1	4	2.0 (2.0)
KTZ	0.125–1	0.5	1	0.6 (0.4)	0.008–4	0.25	1	0.7 (0.9)	0.06–2	0.25	1	0.7(0.8)	0.08–4	0.5	1	0.6 (0.7)
VOR	0.008–0.5	0.008	0.03	0.1 (0.1)	0.008–0.5	0.008	0.03	0.04 (0.1)	0.008–0.06	0.016	0.06	0.03 (0.02)	0.08–0.5	0.008	0.06	0.03 (0.1)
PSZ	0.008–2	0.25	0.5	0.4 (0.5)	0.008–2	0.25	1	0.6 (0.7)	0.008–0.125	0.008	0.125	0.03 (0.05)	0.08–2	0.25	1	0.5 (0.6)
TER	0.008–0.5	0.125	0.125	0.1 (0.1)	0.008–0.5	0.06	0.25	0.1 (0.1)	0.008–0.25	0.008	0.25	0.06 (0.1)	0.08–0.5	0.03	0.25	0.1 (0.1)
FLZ	4–64	16	32	19.7 (16.4)	4–128	32	64	39.5 (38.6)	4–64	8	64	20 (23.6)	4–128	16	64	34.8 (35.4)
GRI	0.125–2	0.5	1	0.8 (0.6)	0.06–2	0.5	1	0.6 (0.4)	0.125–0.5	0.5	0.5	0.4 (0.2)	0.06–2	0.5	1	0.6 (0.5)

^a^ Student’s *t*-test—statistically significant differences (*p* < 0.05) were marked with the same letters. sd: standard deviation. MIC_50_ = MIC at which 50% of the strains were inhibited; MIC_90_ = MIC at which 90% of the strains were inhibited.

**Table 3:** Virulence factors of Low (MIC > MIC_90_) and High (MIC ≤ MIC_90_) drug susceptible strains of *Microsporum canis*. Virulence factors are reported as mean value of phospholipase (Pz), lipase (Lz), hemolytic (Hz) and catalase (Ca; unit = mm) activities, and thermotolerance (GI) with standard deviation (sd) in bracket.

Antifungal Drugs	With Skin Lesions							Without Skin Lesions						
		Pos/Tot (%)	Pz (sd)	Lz (sd)	Hz (sd)	Ca (sd)	GI% (sd)		Pos/Tot (%)	Pz (sd)	Lz (sd)	Hz (sd)	Ca (sd)	GI% (sd)
ITZ	LS MIC > 4	8/74 (11)	0.5 (0.1)	0.7 (0.1)	0.7 (0.05)	43 (13)	53.6 (6.3)	LS MIC > 1	4/26 (15)	0.6 (0.02)	0.5 ^d^ (0.05)	0.7 (0.06)	35 (5)	47.8 (6.2)
	HS MIC ≤ 4	66/74 (89)	0.5 (0.2)	0.8 (0.5)	0.7 (0.1)	46 (11)	54.9 (6.2)	HS MIC ≤ 1	22/26 (85)	0.5 (0.2)	0.7 ^d^ (0.04)	0.5 (0.3)	36 (8)	36.3 (10)
KTZ	LS MIC > 1	4/74 (5.4)	0.5 (0.2)	0.7 (0.3)	0.5 (0.01)	31 (1) ^a^	62.7 (0)	LS MIC > 1	0/26 (0)	0 (0)	0 (0)	0 (0)	0 (0)	0 (0)
	HS MIC ≤ 1	70/74 (9.5)	0.5 (0.2)	0.8 (0.5)	0.7 (0.09)	46 (11) ^a^	54.3 (6.1)	HS MIC ≤ 1	26/26 (100)	0.5 (0.2)	0.7 (0.04)	0.6 (0.3)	35 (8)	38.1 (10.4)
VOR	LS MIC > 0.03	8/74 (11)	0.5 (0.1)	1.5 (1.2)	0.6 (0.06) ^b^	40 (11) ^c^	55.6 (6.9)	LS MIC > 0.03	2/26 (7.7)	0.5 (0)	0.7 (0)	0.7 (0)	40 (0)	38.1 (9.5)
	HS MIC ≤ 0.03	66/74 (89)	0.5 (0.2)	0.7 (0.1)	0.7 (0.09) ^b^	46 (11) ^c^	54.7 (6.2)	HS MIC ≤ 0.03	24/26 (92.3)	0.5 (0.2)	0.7 (0.04)	0.5 (0.3)	34 (8)	36.8 (9.7)
PSZ	LS MIC > 1	10/74 (13.5)	0.4 (0.2)	0.8 (0.04)	0.7 (0.05)	41 (12) ^d^	54.7 (4.2)	LS MIC > 0.5	2/26 (7.7)	0.6 (0)	0.7 (0)	0.8 (0)	40 (0)	41.9 (11.1)
	HS MIC ≤ 1	64/74 (86.5)	0.5 (0.2)	0.8 (0.5)	0.7 (0.09)	46 (12)^d^	54.8 (6.5)	HS MIC ≤ 0.5	24/26 (92.3)	0.5 (0.2)	0.7 (0.04)	0.5 (0.3)	34 (8)	38.4 (10.7)
TER	LS MIC > 0.25	4/74 (5.4)	0.5 (0.05)	0.8 (0.1)	0.7 (0.01)	41 (12) ^e^	61.9 (1.4)	LS MIC > 0.125	1/26 (3.8)	0.6 (0)	0.7 (0)	0.8 (0)	50 (0)	34.6 (0)
	HS MIC ≤ 0.25	70/74 (9.5)	0.5 (0.2)	0.8 (0.5)	0.7 (0.09)	45 (11) ^e^	54.4 (6.2)	HS MIC ≤ 0125	25/26 (96.2)	0.5 (0.2)	0.7 (0.04)	0.5 (0.3)	34 (8)	38.3 (9.0)
FLZ	LS MIC > 64	8/74 (11)	0.4 (0.3)	0.7 (0.03)	0.6 (0.1) ^f^	47 (11)	59.7 (4.3)	LS MIC > 32	2/26 (7.7)	0.6 (0)	0.7 (0)	0 (0)	48 (0)	34.6 (0)
	HS MIC ≤ 64	66/74 (89)	0.5 (0.2)	0.8 (0.5)	0.7 (0.1) ^f^	45 (11)	53.4 (6.2)	HS MIC ≤ 32	24/26 (92.3)	0.5 (0.2)	0.7 (0.04)	0.6 (0.3)	37 (4)	38.4 (10.7)
GRI	LS MIC > 1	2/74 (2.7)	0.4 (0)	0.8 (0)	0.5 (0)	60 (0)	52.9 (0)	LS MIC > 1	4/26 (15.4)	0.7 (0.1)	0.7 (0.05)	0.7 (0.03)	27 (3) ^g^	26.2 (0)
	HS MIC ≤ 1	72/74 (97.3)	0.5 (0.2)	0.8 (0.4)	0.7 (0.09)	45 (11)	54.8 (6.3)	HS MIC ≤ 1	22/26 (84.6)	0.5 (0.2)	0.7 (0.04)	0.5 (0.3)	36 (7) ^g^	40.2 (10)

^a–g^ Student’s *t*-test—statistically significant differences (*p* < 0.05) were marked with the same letters. For Pz, Lz and Hz, lower value represents higher activity.

## Data Availability

Publicly available datasets were analyzed in this study. This data can be found here: [Accession numbers: LR989561-LR989562].

## Supplementary Materials

The following are available online at https://www.mdpi.com/2079-6382/10/3/296/s1, Table S1: title: Raw data and genotypes; Figure S1: Virulence factors.
