# Supplementary material for: Virulence and Antifungal Susceptibility of Microsporum canis Strains from Animals and Humans
Source: Antibiotics (Basel). 2021 Mar 12;10(3):296. doi: 10.3390/antibiotics10030296 (PMC8000290; doi:10.3390/antibiotics10030296)
Supplement: Supplementary file 1 [file antibiotics-10-00296-s001.pdf]

antibiotics

MDPI

Article

Virulence and Antifungal Susceptibility of *Microsporum canis* Strains from Animals and Humans

Chioma Inyang Aneke [1,2], Wafa Rhimi [1], Vit Hubka [3,4], Domenico Otranto [1,5] and Claudia Cafarchia [1,*]

[1] Dipartimento di Medicina Veterinaria, Università degli Studi "Aldo Moro", 70010 Bari, Italy; chioma.aneke@unn.edu.ng (C.I.A.); wafa.rhimi@uniba.it (W.R.); domenico.otranto@uniba.it (D.O.)
[2] Department of Veterinary Pathology and Microbiology, University of Nigeria, 410001 Nsukka, Nigeria
[3] Department of Botany, Faculty of Science, Charles University, 12801 Prague 2, Czech Republic; vit.hubka@gmail.com (V.H.)
[4] Laboratory of Fungal Genetics and Metabolism, Institute of Microbiology of the Academy of Sciences, 14220 Prague 4, Czech Republic
[5] Faculty of Veterinary Sciences, Bu-Ali Sina University, 6517658978 Hamedan, Iran
* Correspondence: claudia.cafarchia@uniba.it; Tel.: +39-080-4679834; +39-3385257824

Table S1. Raw data and genotypes.

Sample Code	Group (A, AL, H)	Phospholipase	Low Catalase Producer	High Catalase Producer	Lipase	Hemolysis	Low Thermotolerance	High Thermotolerance	ITS Genotype
CD1134	A	Y		Y	Y	Y	Y		G1
CD1598	AL	Y		Y	Y	Y	Y		G1
CD1192	A	Y		Y	Y	Y	Y		G1
CD1193	AL	Y		Y	Y	Y	Y		G1
CD1209	AL	Y		Y	Y	Y		Y	G1
CD1306	AL	Y		Y	Y	Y	Y		G1
CD387	AL	Y		Y	Y	Y	Y		G1
CD409	A	N		Y	Y	Y	Y		G1
CD412	AL	Y		Y	Y	Y	Y		G1
CD430	AL	Y		Y	Y	Y	Y		G1
CD760	A	Y		Y	Y	Y		Y	G1
CD1307	AL	Y		Y	Y	Y		Y	G2
CD1308	A	Y		Y	Y	Y	Y		G2
CD1289	AL	Y		Y	Y	Y	Y		G2
CD1131	AL	Y		Y	Y	Y		Y	G1
CD1132	AL	Y		Y	Y	Y	Y		G1
CD1135	AL	Y	Y		Y	Y	Y		G1
CD1143	H	Y		Y	Y	Y	Y		G1
CD1145	A	Y		Y	Y	N		Y	G1
CD1146	AL	Y		Y	Y	Y		Y	G1
CD1148	AL	Y		Y	Y	Y	Y		G1
CD1149	AL	Y		Y	Y	Y		Y	G1
CD1150	H	Y		Y	Y	Y	Y		G1
CD1151	AL	N	Y		Y	Y		Y	G1
CD1152	H	Y		Y	Y	Y	Y		G1
CD1153	AL	N		Y	Y	Y	Y		G1
CD1190	A	Y	Y		Y	N	Y		G1
CD1191	AL	Y		Y	Y	Y		Y	G1
CD1194	AL	Y		Y	Y	Y	Y		G1
CD1195	AL	Y		Y	Y	Y		Y	G1
CD1196	H	Y		Y	Y	Y	Y		G1
CD1211	AL	Y		Y	Y	Y		Y	G1
CD1229	AL	N	Y		Y	Y	Y		G1
CD1230	AL	Y		Y	Y	Y	Y		G1
CD1231	A	Y		Y	Y	Y		Y	G1
CD1232	AL	Y	Y		Y	Y	Y		G1
CD1233	AL	Y		Y	Y	Y		Y	G1
CD1235	AL	Y		Y	Y	Y		Y	G1

CD1242	AL	Y		Y	Y	Y		Y	G1
CD1279	AL	Y		Y	Y	Y	Y		G1
CD1320	AL	Y	Y		Y	Y	Y		G1
CD1565	AL	Y		Y	Y	Y	Y		G1
CD1567	AL	Y		Y	Y	Y	Y		G1
CD1595	A	Y		Y	Y	Y	Y		G1
CD1600	AL	Y		Y	Y	Y		Y	G1
CD1601	AL	Y		Y	Y	Y	Y		G1
CD1602	AL	Y		Y	Y	Y		Y	G1
CD367	AL	Y		Y	Y	Y	Y		G1
CD382	AL	Y		Y	Y	Y		Y	G1
CD383	A	Y		Y	Y	Y	Y		G1
CD384	H	Y		Y	Y	Y	Y		G1
CD396	H	Y		Y	Y	Y		Y	G1
CD415	A	Y		Y	Y	Y		Y	G1
CD416	AL	Y		Y	Y	Y		Y	G1
CD441	AL	Y	Y		Y	Y	Y		G1
CD448	A	Y	Y		Y	Y		Y	G1
CD761	AL	N		Y	Y	Y		Y	G1
CD975	AL	Y		Y	Y	Y	Y		G1
CD976	AL	Y		Y	Y	Y	Y		G1
CD978	A	N		Y	Y	N		Y	G1
CD979	AL	Y		Y	Y	Y		Y	G1
CD980	AL	N		Y	Y	Y	Y		G1

Key: A = Animal without lesion; AL = Animal with lesion, H = Human; Y = Yes; N = No.

Figure S1. Virulence factors. (**a**) Phospholipase activity on egg-yolk medium—a clear halo zone of clearance around the colony indicated phospholipase production. (**b**) Lipase activity on 1% tween 80 medium—a clear halo zone of clearance around the colony indicated lipase production (**c**) Catalase activity on PDA medium—column of bubbles indicated catalase production. (**d**) Hemolytic activity on Columbia blood agar—a transparent zone of clearance around the colony indicated hemolysis. (**e**) Thermotolerance determination on PDA medium.

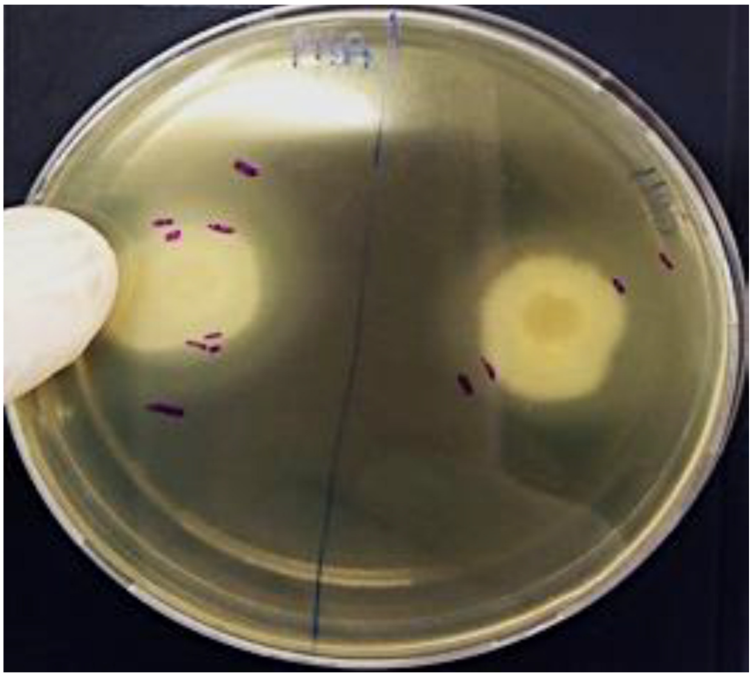

(a)

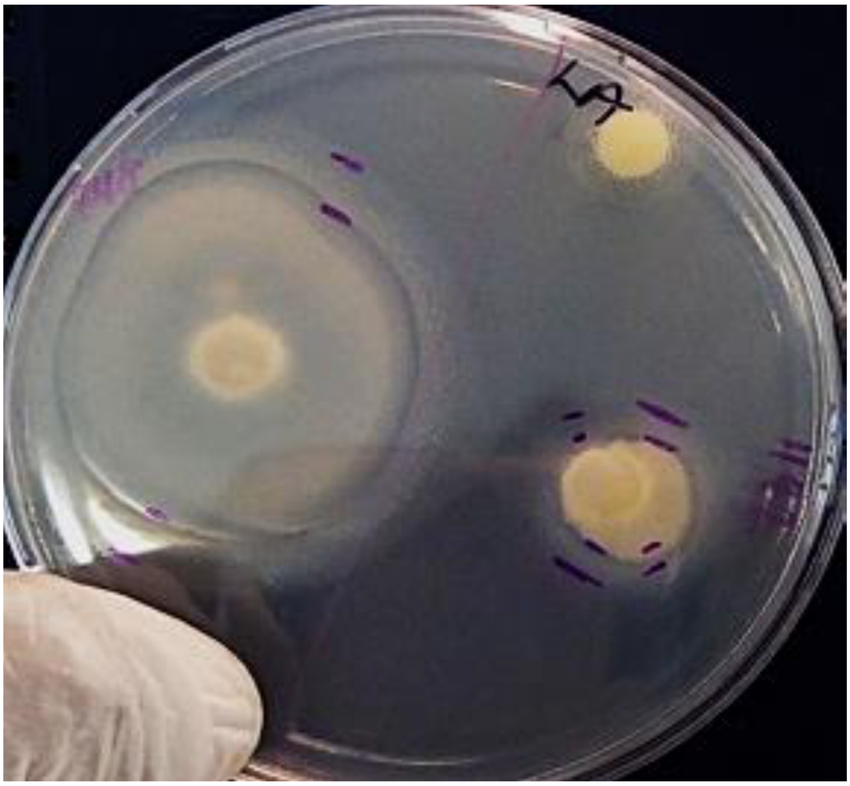

(b)

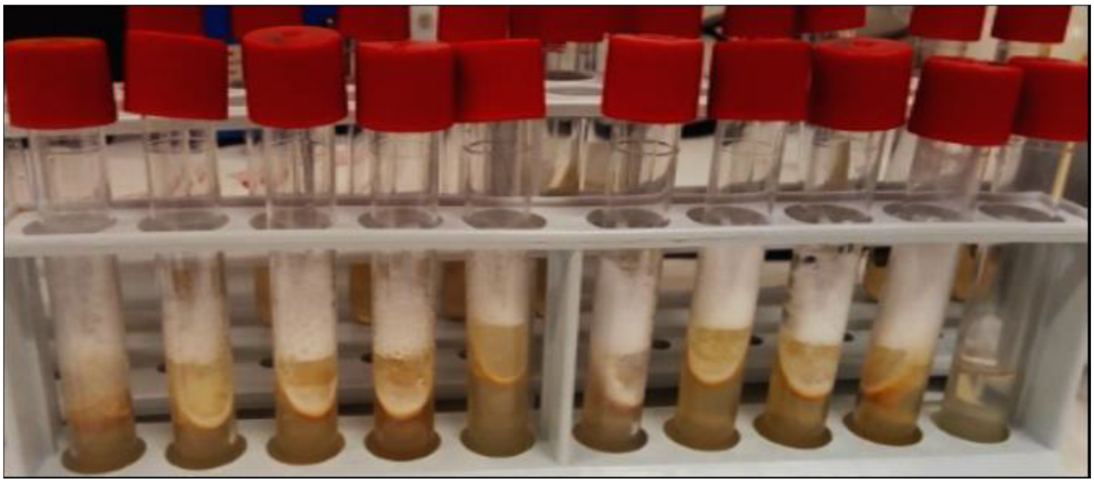

(c)

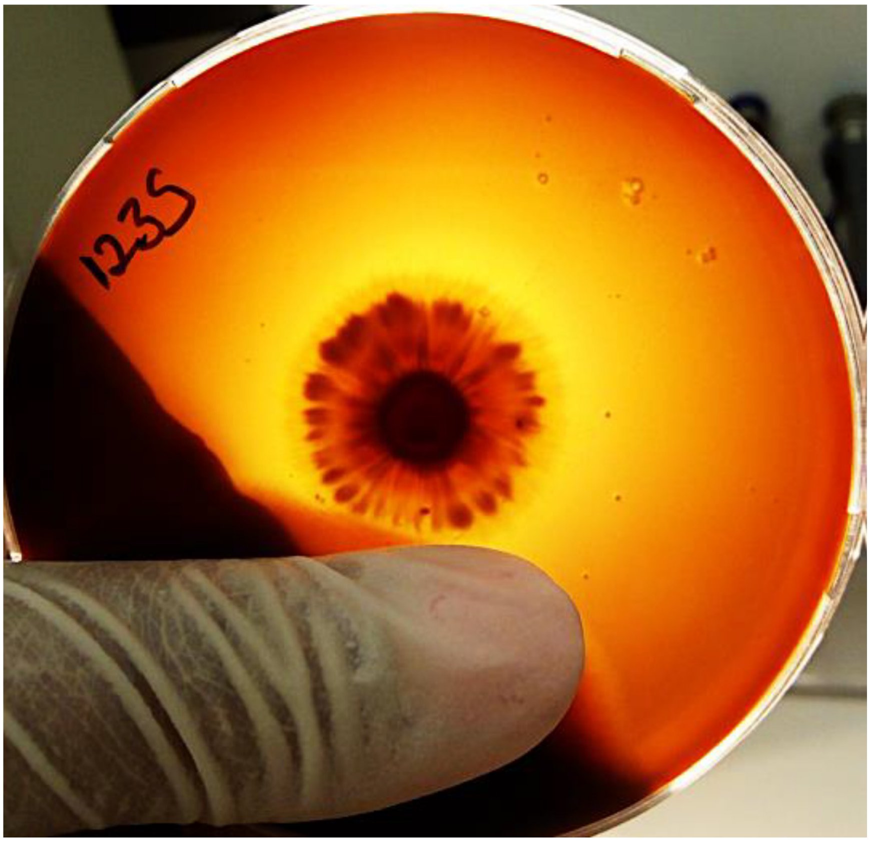

(d)

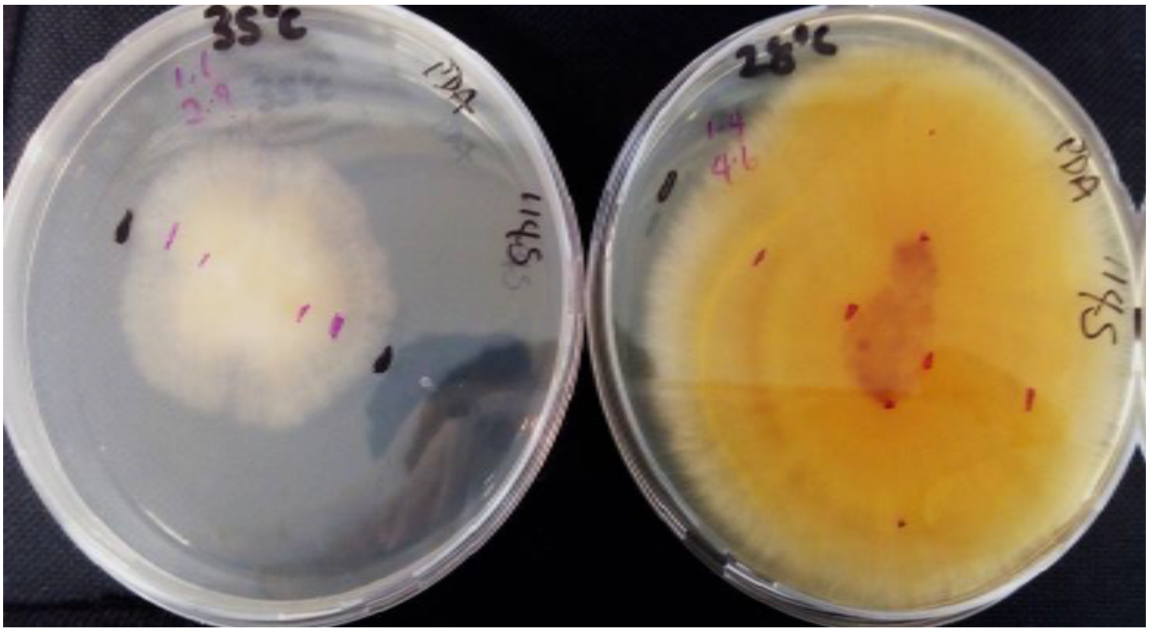

(e)
